# Heat-Killed *Enterococcus faecium* KU22001 Having Effective Anti-Cancer Effects on HeLa Cell Lines at a Lower Temperature

**DOI:** 10.4014/jmb.2310.10050

**Published:** 2024-01-08

**Authors:** Jun-Su Ha, Na-Kyoung Lee, Hyun-Dong Paik

**Affiliations:** Department of Food Science and Biotechnology of Animal Resources, Konkuk University, Seoul 05029, Republic of Korea

**Keywords:** *Enterococcus faecium*, paraprobiotics, anti-cancer effect, cervical cancer, exopolysaccharide

## Abstract

The anti-cancer effects of heat-killed *Enterococcus faecium* KU22001 (KU22001), KU22002, and KU22005 isolated from human infant feces were investigated. The anti-proliferative activity of these strains against various cancer cell lines was evaluated using the MTT assay. To determine the production of exopolysaccharides (EPS) with potential anti-cancer effect, ethanol precipitation and phenol-sulfuric acid method was used with the cell free supernatant of strains grown at 25°C or 37°C. The EPS yield of *E. faecium* strains was higher at 25°C than at 37°C. Among these *E. faecium* strains, KU22001 grown at 25°C was associated with the highest *bax*/*bcl-2* ratio, effective apoptosis rate, cell cycle arrest in the G_0_/G_1_ phase, and condensation of the nucleus in the cervical cancer HeLa cell line. In conclusion, these results suggest that KU22001 can be beneficial owing to the anti-cancer effects and production of functional materials, such as EPS.

## Introduction

Enterococci are lactic acid bacteria (LAB) that find extensive applications in various dairy products, playing vital roles such as initiation cultures for development of food supplements and functional foods [1, 2]. Among the *Enterococcus* species, *Enterococcus faecium* is one of the most commonly encountered species in human feces, along with *Enterococcus faecalis* [3]. Nevertheless, owing to the contrasting characteristics of different enterococcal strains, these bacteria have emerged as one of the most contentious categories of LAB [4-6]. Consequently, to mitigate potential risks, the focus of research is progressively transitioning from probiotics to inactivated bacterial cells, collectively known as paraprobiotics [7, 8]. The use of paraprobiotics have been used for healthy foods to humans and animals are also known or considered as nonlive microbial cells, crude cell extracts, or ghost probiotics [9]. Along with the increase in the interest in paraprobiotics as a category of pharmaceuticals, research on cancer prevention and treatment has continued [10]. Paraprobiotics comprise dead microbial cell components, such as teichoic acids, peptidoglycan-derived mucopeptides, exopolysaccharides, moonlight proteins, pili proteins, and cell wall-bound biosurfactants [11-13].

Exopolysaccharides (EPS) are extracellular surface carbohydrate polymers that can loosely bind to the bacterial cell surface or be released into the surrounding cell environment [14]. Some LAB produce higher yields of EPS at suboptimal temperatures than at optimal growth temperatures [15, 16]. *E. faecium* AK1247 shows the maximum EPS production when incubated at 25°C than that at 30°C or 37°C [17]. Low temperatures lead to increased environmental stress in cells, allowing them to produce more EPS to protect themselves from detrimental environmental conditions [18]. In addition, EPS extracted from LAB have shown anti-proliferative effects on a variety of cancer cells, such as those associated with the cancer development in the intestine, liver, and cervix [19, 20]. Both intracellular and extracellular signals activate the apoptotic pathway, and two different intrinsic and extrinsic pathways correlate with the signal type. The intrinsic pathway of apoptosis is regulated by the B-cell lymphoma-2 (*bcl-2*) protein family, comprising the proapoptotic *bcl-2*-associated X protein (*bax*) and antiapoptotic *bcl-2* proteins. Furthermore, apoptosis is induced by caspases, a class of cysteine proteases that cleave specific target proteins such as *caspase-9*, the initiator caspase, and *caspase-3*, the executioner caspase [21].

This study examined the anti-cancer effects of heat-killed *E. faecium* KU22001, *E. faecium* KU22002, and *E. faecium* KU22005 isolated from human infant feces. In addition, the mechanism of the anti-cancer effect of heat-killed *E. faecium* strains was examined based on the expression of apoptotic genes, flow cytometry analysis, and 4',6-diamidino-2-phenylindole (DAPI) staining results corresponding to specific incubation temperatures.

## Material and Methods

### Strains, Culture Media, and Reagents

*E. faecium* KU22001, *E. faecium* KU22002, and *E. faecium* KU22005 were isolated from human infant feces of different healthy individuals, provided by the Korea Research Institute of Bio medical Science (KRIBS, Republic of Korea). *Lacticaseibacillus rhamnosus* GG was obtained from the Korean Collection for Type Cultures (KCTC, Republic of Korea) and used as the reference strain. Samples were cultured in de Man, Rogosa, and Sharpe medium (MRS; BD Biosciences, USA). Roswell Park Memorial Institute (RPMI) 1640 medium, Dulbecco’s modified Eagle’s medium (DMEM), penicillin/streptomycin (P/S), fetal bovine serum (FBS), and phosphate-buffered saline (PBS) were acquired from HyClone (USA). All other reagents were purchased from Sigma-Aldrich (USA).

### Culture Conditions and Sample Preparation

LAB strains were incubated in the MRS broth at 25°C or 37°C for 20 h. All cultures were centrifuged at 14,240 ×*g* for 5 min at 4°C, cleaned twice, and suspended in PBS. The heat-killed cells were additionally exposed to high temperatures at 90°C for 30 min in a water bath, centrifuged at 14,240 ×*g* for 5 min at 4°C, and diluted in growth medium. The growth medium without bacteria was used as a negative control.

### Cell Cultures

MRC-5 (human lung cell line, KCLB 10171), RAW 264.7 (murine macrophage cell line KCLB 40071), AGS (human stomach adenocarcinoma cell line, KCLB 21739), HT-29 (human colon adenocarcinoma cell line, KCLB 30038), DLD-1 (human colon adenocarcinoma cell line, KCLB 10221), LoVo (human colon adenocarcinoma cell line, KCLB 10229), Caco-2 (human colon adenocarcinoma cell line, KCLB 30037), HeLa (human cervix adenocarcinoma cell line, KCLB 1002), MCF-7 (human breast adenocarcinoma cell line, KCLB 30022), A549 (human lung adenocarcinoma cell line, KCLB 10185), and HepG2 cells (human liver adenocarcinoma cell line, KCLB 88065) were obtained from the Korean Cell Line Bank (KCLB; Seoul National University, Republic of Korea). The cell lines were cultured in RPMI 1640 (AGS, DLD-1, LoVo, HT-29, HeLa, MCF-7, and A549 cells) or DMEM (MRC-5, Caco-2, and HepG2 cells) as cell-dependent media containing 10% FBS and 1% P/S at 37°C in an atmosphere of 5% CO_2_ and 95% air.

### Anti-Proliferative Activity

The anti-proliferative activity of various cancer cell lines was tested utilizing the MTT assay [22]. Cells were planted in 96-well plates and cultured overnight. The cells were dealt with the samples (8 and 9 log CFU/ml) and cultured for 48 h. Next, the cells were washed twice with PBS, treated with 100 μl of MTT reagent (0.5 mg/ml), and reacted for 4 h. The MTT reagent was then eliminated, and 150 μl of dimethyl sulfoxide (DMSO) was included. Absorbance was assessed at 570 nm, and cytotoxicity was determined as follows:

Cytotoxicity (%) = (1-A_sample_/A_control_) × 100

where A_sample_ and A_control_ indicate the absorbance values of the treated and control samples, respectively.

### EPS Analysis Using the Phenol-Sulfuric Acid Method

EPS concentrations were determined using ethanol precipitation [23]. The cell liberated supernatants were collected by centrifugation at 14,240 ×*g* at 4°C for 5 min. The EPS was precipitated from the supernatant with three quantities of cold ethanol (95% purity) at 4°C overnight, and gathered by centrifugation at 14,240 ×*g* for 20 min. The EPS pellets were dissolved in distilled water. Quantitative analysis of EPS yield was performed using the phenol-sulfuric acid method [24]. First, 500 μl of considered samples were added with 500 μl of 4% phenol afterward the addition of 2.5 ml of sulfuric acid (96% purity) into all the tubes. The solutions were analyzed at 490 nm using a UV spectrophotometer, with distilled water as the blank and glucose as the standard. The absorbance values of the measured samples were adjusted with distilled water below 1.0. Finally, the EPS concentrations present in the samples were determined based on a graph plotting the absorbance against the EPS calibration standards.

### RNA Extraction and Semi-Quantitative Real-Time PCR

Semi-quantitative real-time PCR was performed to evaluate the expression of apoptosis-related genes in HeLa cells. HeLa cells were seeded at a density of 1 × 10^6^ cells/well in 6-well plates and incubated for 24 h. Subsequently, 1 ml of the heat-killed LAB sample was added, followed by incubation for 24 h. Total RNA was isolated from cells using the RNeasy Mini Kit (Qiagen, Germany), and the cDNA synthesis Kit (Thermo-Fisher Scientific, USA) was used for cDNA synthesis according to the manufacturer's instructions. The expression of apoptosis-related genes (*bax*, *bcl-2*, *caspase-3*, and *caspase-9*) was determined using SYBR Green PCR Master Mix by semi-quantitative real-time PCR (PikoReal 96; Scientific Pierce, USA). The β-actin housekeeping gene was used as a control. The primers used are listed in Supplementary Table 1S [25].

The PCR conditions are described as follows: 94°C for 2min, afterward 35 cycles at 94°C for 15s, 55°C for 30s, annealing at 68°C for 60s, and a final extension at 72°C for 5min. The outcomes were analyzed using the delta–delta Cq method. A melting curve was used to investigate reaction specificity.

### Flow Cytometry Analysis of Apoptosis

Cell apoptosis was evaluated using the fluorescein isothiocyanate (FITC) Annexin V/Dead Cell Apoptosis Kit (Thermo Fisher Scientific), as per the manufacturer’s instructions. HeLa cells were seeded in 6-well plates at a density of 1 × 10^6^ cells/well, and the plates were dealt with the heat-killed LAB samples for 48 h. After treatment, the cells were harvested by trypsinization, cleaned through PBS, suspended in 1 × annexin binding buffer, and dyed with annexin V-FITC and propidium iodide (PI) solution for 20 min in the dark. Subsequently, the dyed cells were resuspended in 1 × annexin binding buffer and planned to monitor apoptosis [26]. FITC and PI were identified in the FL-1 and FL-2 channels, respectively using CytoFLEX (Beckman Coulter, USA).

### Flow Cytometry Analysis of Cell Cycle Distribution

The cell cycle was analyzed according to a previously described protocol [27]. HeLa cells were seeded in 6-well plates and treated with heat-killed LAB for 48 h. Adherent cells were washed with PBS, and trypsin was added for 3 min to detach the cells. After centrifugation at 350 ×*g* at 4°C for 5 min, the cell pellet was washed once with ice-cold PBS and gently vortexed with 3 ml cold 70% ethyl alcohol at 4°C for 2 h. After incubation, the cell pellet was washed twice with ice-cold PBS by centrifuging the sample at 14,240 ×*g* at 4°C for 3 min. Finally, 100 μg/ml RNase A and 50 μg/ml propidium iodide (PI) staining solution was added to the samples for 1 h at room temperature in the dark. The samples were analyzed using CytoFLEX, and the results were analyzed using CytExpert 2.5.0.77 software (Backman Counter, USA).

### DAPI Staining and Fluorescence Microscopy

DAPI staining was used to visually examine the indicators of apoptotic cells. HeLa cells were seeded at a density of 1 × 10^4^ cells in confocal dishes and incubated for 24 h. Next, heat-killed LAB samples were added to the cells, followed by incubation for 48 h. Last in order of the incubation period, the cells were washed twice with PBS. The cells were then covered with 1 μg/ml of DAPI working solution and incubated for 10 min at room temperature. Lastly, the stained cells were washed with PBS, and fluorescence microscopy was carried out using a super-resolution confocal laser scanning microscope (Carl Zeiss LSM 800, Germany) [28].

### Statistical Analysis

Represented data are presented as the mean ± standard deviation of three repetitions. One-way analysis of variance (ANOVA) was applied to confirm significant differences. The mean values were used for Duncan’s multiple range test for post-hoc verification (*p < 0.05 and **p < 0.01). SPSS (IBM Corp., USA) was used for statistical analysis.

## Results and Discussions

### Anti-Proliferative Activity

The cytotoxicity of the heat-killed LAB strains against normal and cancer cells was measured using the MTT assays. The viability of normal MRC-5 and RAW264.7 cells dealt with these samples was better than 90%. Thus, the three heat-killed *E. faecium* strains were considered non-toxic to normal cells (Supplementary data in Table 2S). Conversely, as shown in Table 1, an antiproliferative effect was observed on all cancer cells, except HT-29 cells, at 9 log CFU/ml (> 20% cytotoxicity). *E. faecium* KU22001 showed better or similar anti-proliferative effects than *E. faecium* KU22002 and *E. faecium* KU22005, except in AGS, HT-29, and DLD-1 cells.

**Fig. 1:**
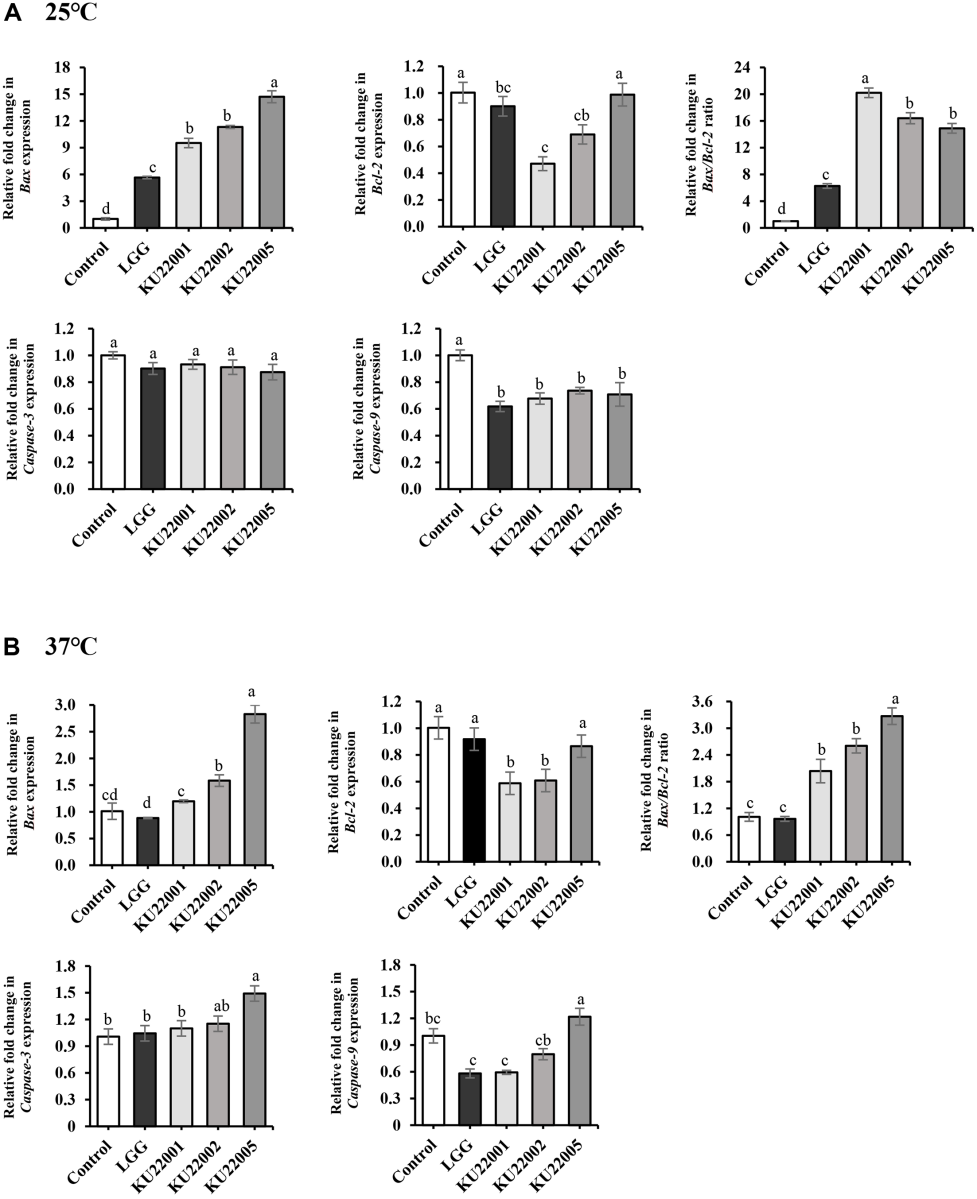
The expression of apoptosis-associated genes in HeLa cells by heat-killed LAB strains. (**A**) Treatment with 9 log CFU/ml of LAB strains grown at 25°C. (**B**) Treatment with 9 log CFU/ml of LAB strains grown at 37°C. LGG, *Lacticaseibacillus rhamnosus* GG; KU22001, *Enterococcus faecium* KU22001; KU22002, *Enterococcus faecium* KU22002; KU22005, *Enterococcus faecium* KU22005. The data of relative mRNA expression are represented as the mean ± SD of three experiments. Different letters above the value indicate significant differences for each characteristic (*p* < 0.05).

Table 2 and Fig. 1 show the results of cytotoxicity and the expression of apoptosis-associated genes in HeLa cells according to the culture conditions, namely duration and temperature. The HeLa cells were treated heat-killed LAB at 9 log CFU/ml. The cells treated with heat-killed LAB strains grown at 25°C showed significantly increased cytotoxic effects compared to those treated with heat-killed LAB strains grown at 37°C.

### EPS Production

EPS can exert anti-cancer effects by modulating tumor development via various mechanisms, including promotion of apoptosis and induction of cell cycle arrest [29, 30]. EPS production by the LAB strains was decided on using the phenol-sulfuric acid method. Table 3 shows that the *E. faecium* strains grown at 37°C showed more than 1 mg/ml of EPS production, which was higher than that shown by LGG. Additionally, the EPS production of LAB strains cultured at 25°C increased remarkably compared to that of the strains grown at 37°C. Of these, *E. faecium* KU22001 strain showed the highest production. These results depend on EPS form to vary sugar composition, molecular weight, and linkage type between polysaccharides. Mainly, anti-cancer polysaccharides contain high amounts of mannose in sugar composition and tend to show high molecular weight or β-1,3-linke [31-34]. Therefore, further experiments are needed to determine which LAB strains have the remarkable anti-cancer activity.

### RNA Extraction and Semi-Quantitative Real-Time PCR

RT-PCR data showed that heat-killed LAB strains regulated the expression of apoptotic genes. HeLa cells were selected because of their high *bax*/*bcl-2* ratios. While the *bax*/*bcl-2* ratio of cells treated with all heat-killed LAB increased significantly, *E. faecium* KU22001 cultured at 25°C showed the highest vales (Fig. 1). However, among the *E. faecium* strains cultured at 37°C, *E. faecium* KU22005 group showed highest values of *bax*/*bcl-2* ratio and expression of *caspase-3* and *caspase-9*. The turnaround of the *bax*/*bcl-2* ratio associated with *E. faecium* KU22001 and *E. faecium* KU22005 under different culture temperature conditions can be attributed to the growth characteristics of these strains.

*E. faecium* KU22001 grown at the optimal growth temperature of 37°C had a higher log CFU/ml value than *E. faecium* KU22005 (Fig. S1). However, *E. faecium* KU22001 grown at 25°C had a lower from 9.0 to 9.1 log CFU/ml, and KU22005 showed no difference. This is because the slow growth of *E. faecium* KU22001 cultured at 25°C may decrease the rate of cell wall synthesis, thereby providing more isoprenoid lipid carrier precursor molecules for the synthesis of EPS [35]. Interestingly, in contrast to the high *bax*/*bcl-2* ratio, the *caspase-3* and *caspase-9* expression levels in HeLa cells treated with heat-killed LAB strains grown at 25°C was not statistically different from those of the control and did not show an increase. This related mechanisms may involve other initiator and effector of caspases, or mitochondrial outer membrane permeabilization (MOMP), an important step in the intrinsic cell death pathway regulated by *bax* and *bcl-2*, often induces apoptosis through various intermembrane-space proteins such as apoptosis-induing factor (AIF) and endonuclease G regardless of the caspase activity [36, 37]. This other caspase-independent form of cell death is most likely related to the wide properties of MOMP. Here, all mitochondria often undergo permeabilization, cause a ongoing and tremendous loss of mitochondrial function [38].

### Apoptosis Assay

**Fig. 2:**
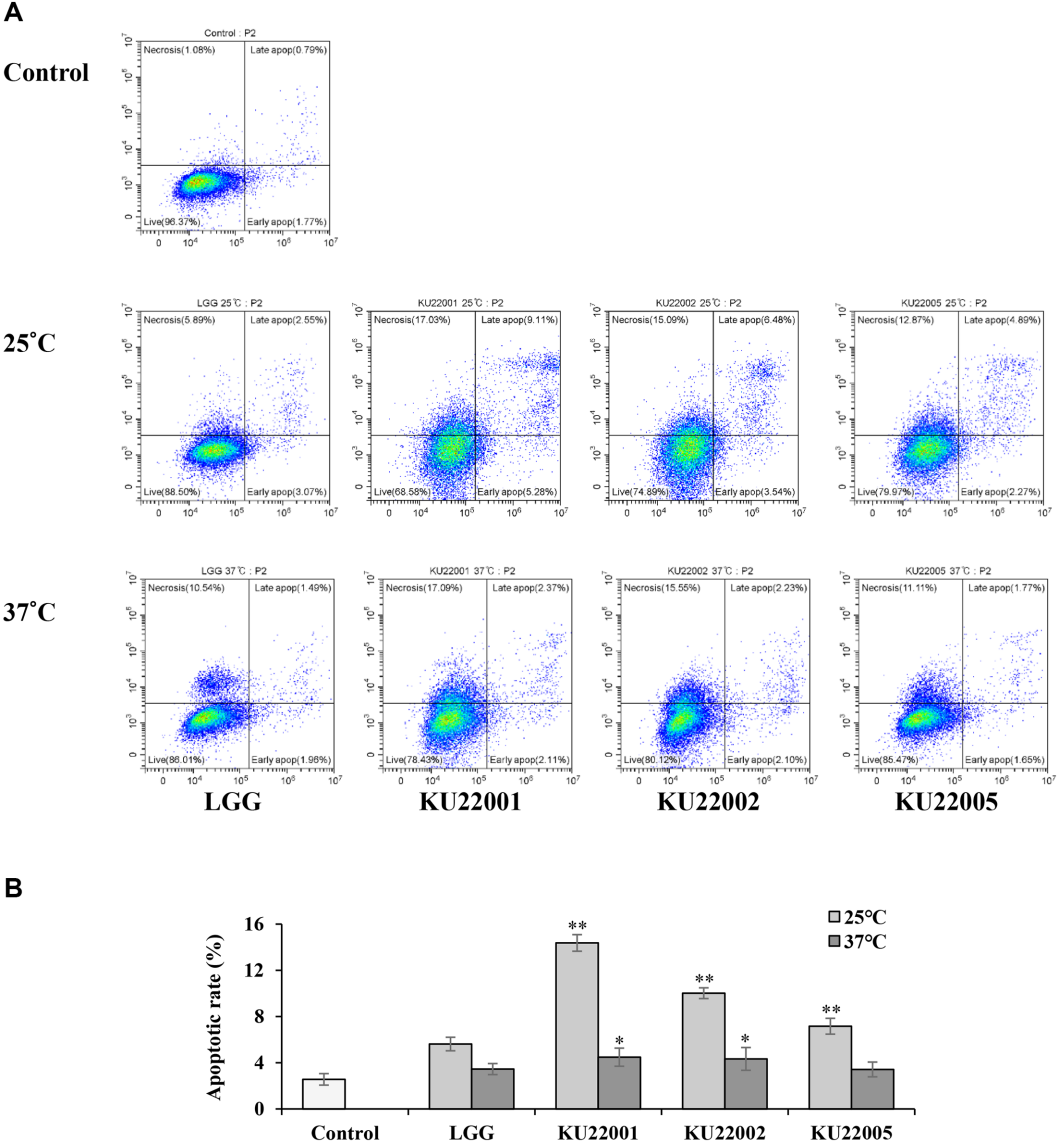
Apoptosis of HeLa cells treated with heat-killed LAB strains grown at 25°C and 37°C determined using flow cytometry. (**A**) Representative Annexin V-FITC/PI staining for apoptosis. (**B**) Apoptotic rate (%). LGG, *Lacticaseibacillus rhamnosus* GG; KU22001, *Enterococcus faecium* KU22001; KU22002, *Enterococcus faecium* KU22002; KU22005, *Enterococcus faecium* KU22005. Results are presented as mean ± SD derived from three independent experiments. *p < 0.05, and **p < 0.01 compared with the control.

Apoptotic death in HeLa cells was identified by double staining with Annexin V-FITC and PI, succeeded to flow cytometric analysis. As shown in Fig. 2, cell populations were divided into necrotic (upper left, UL), late apoptotic (upper right, UR), live (lower left, LL), and early apoptotic (lower right, LR) quadrants. The apoptosis rate, which reflects the sum of early apoptosis (LR) and late apoptosis (UR), was observed. Treatment with heat-killed LAB strains grown at both 25°C and 37°C compared to the control, so that after a significant increase in apoptotic rate in 25°C than 37°C. For treatment with *E. faecium* KU22001, which showed the highest apoptosis rate, a remarkable increase in the proportion of apoptotic cells from 4.48% to 14.39% was noted at both 25°C and 37°C, respectively.

### Cell Cycle Analysis

**Fig. 3:**
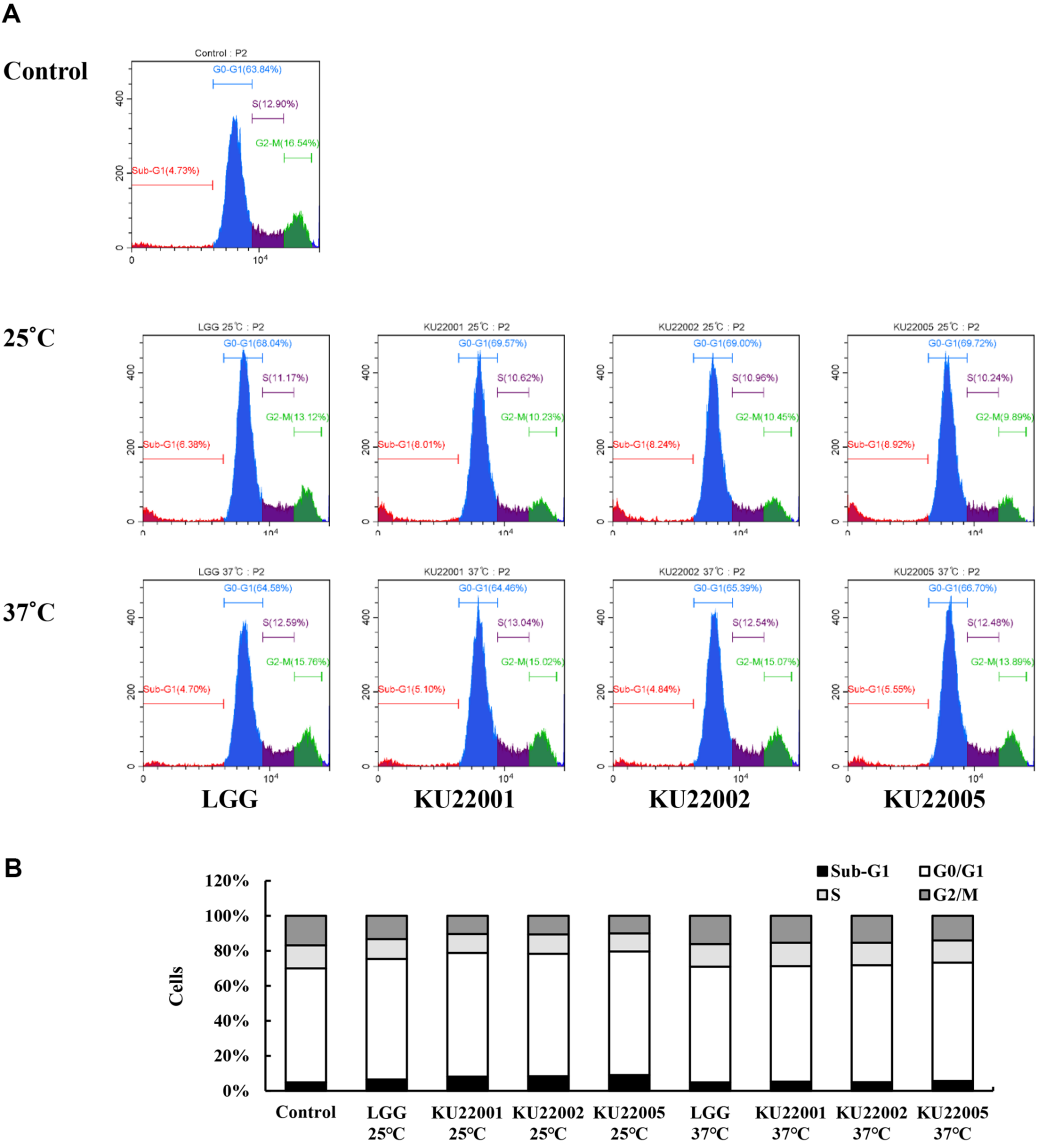
Cell cycle of HeLa cells treated with heat-killed LAB strains grown at 25°C and 37°C determined using flow cytometry. (**A**) Cell cycle distribution. (**B**) Flow cytometry analysis. HeLa cells were treated with heat-killed LGG and *E. faecium* for 48 h. The percentages of cells in each phase were analyzed by CytExpert 2.5.0.77 software (Backman Counter).

Cell cycle arrest and apoptosis are common mechanisms that regulate cell proliferation [39]. PI-flow cytometric analysis was used to investigate the change in DNA content throughout the cell cycle progression following treatment with heat-killed LAB strains. As shown in Fig. 3, treatment of HeLa cells with heat-killed LAB strains grown at 25°C resulted in a higher proportion of cells in the G_0_/G_1_ phase, which corresponds to apoptosis. In addition, the percentage of cells in the sub-G_1_ phase significantly increased compared to that observed with the control and treatment with strains at 37°C, reaching 8.01%, 8.24% and 8.92% for KU22001, KU22002 and KU22005, respectively.

### Morphological Changes of Heat-Killed LAB Strains Using DAPI Staining

**Fig. 4:**
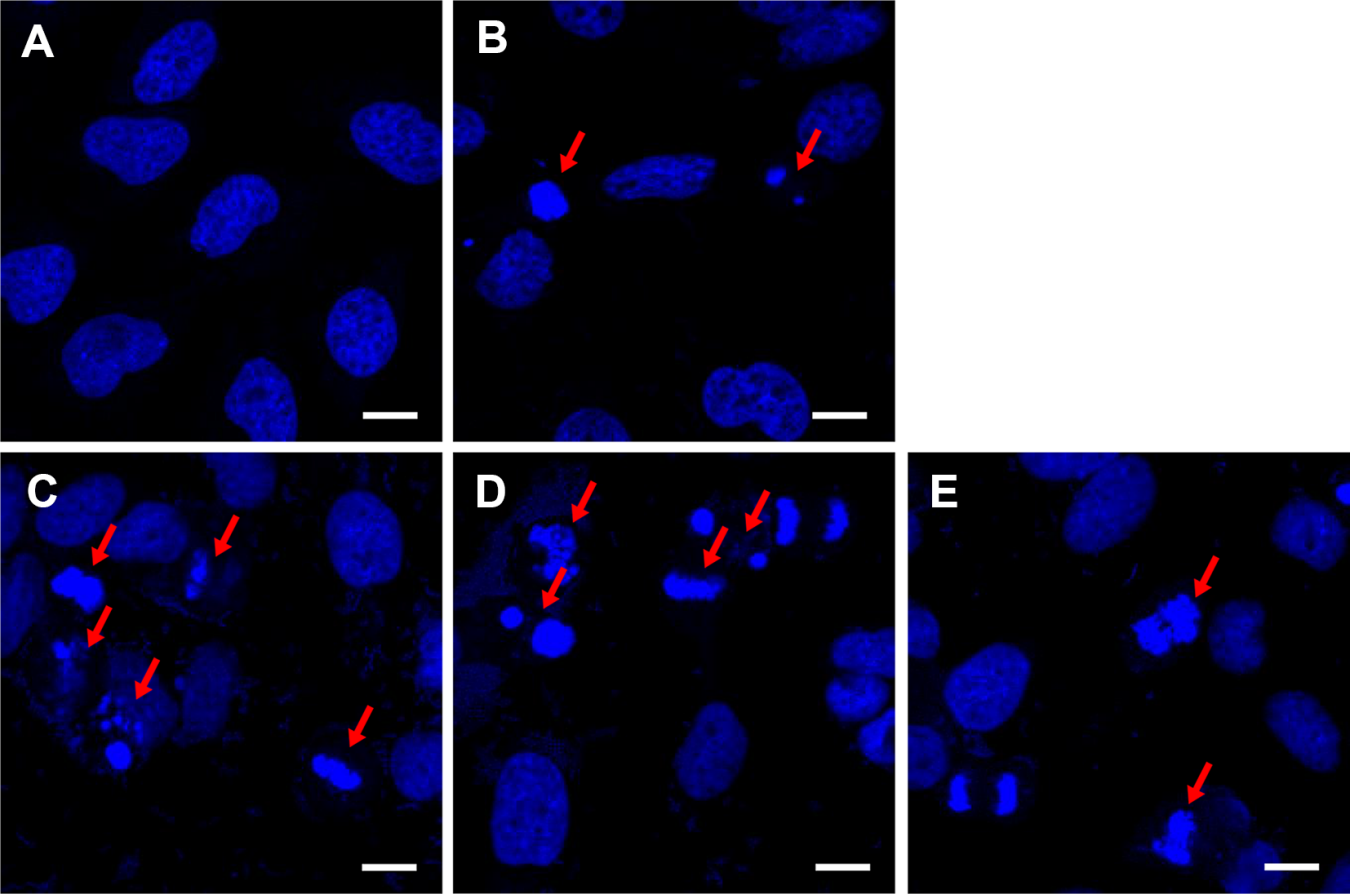
Morphological changes in HeLa cells with heat-killed LAB strains grown at 25°C detected using confocal imaging. (**A**) Control, (**B**) LGG, (**C**) KU22001, (**D**) KU22002, and (**E**) KU22005. The red arrow shows the condensation or breakage of the nucleus of treated cells; white scale bar indicates 10 μm. LGG, *Lacticaseibacillus rhamnosus* GG; KU22001, *Enterococcus faecium* KU22001; KU22002, *Enterococcus faecium* KU22002; KU22005, *Enterococcus faecium* KU22005.

Morphological changes in HeLa cells treated with heat-killed LAB strains were assessed using DAPI staining and confocal imaging. As shown in Fig. 4, the control group had normal elongated nuclei with well-distributed chromatin. In contrast, treatment with heat-killed LAB strains cultured at 25°C showed apoptotic morphology, including condensed and fragmented chromatin. The released AIF from mitochondria induces apoptosis by chromatic condensation and margination [40]. The degree of change in cell morphology was higher corresponding to *E. faecium* strains than that observed with LGG treatment. The observed morphological changes in the nuclei confirmed that *E. faecium* strains had the capacity to induce apoptosis in HeLa cells.

In conclusion, *E. faecium* KU22001, *E. faecium* KU22002, and *E. faecium* KU22005 isolated from human infant feces were investigated for their anti-cancer effects. These heat-killed *E. faecium* strains exhibited selective antiproliferative effects on various cancer cells in a dose-dependent manners, with non-toxic effects on normal fibroblast cells. In addition, *E. faecium* strains grown at 25°C exhibited a more potent anti-cancer effect against HeLa cells than those grown at 37°C, which is the optimal growth temperature. Particularly, *E. faecium* KU22001 grown at 25°C showed the highest EPS production and promotion of the upregulation of the expression of *bax* and downregulation of the expression of the *bcl-2* compared to control cells. Flow cytometric assessments and fluorescent microscopic observation showed that *E. faecium* KU22001 reduced the viability of HeLa cells by inducing apoptosis and cell cycle arrest in the G_0_/G_1_ phase. These results suggest that heat-killed *E. faecium* KU22001 can be used as a prophylactic functional food with anti-cancer effects.

## Supplemental Materials

Supplementary data for this paper are available on-line only at http://jmb.or.kr.



## Figures and Tables

**Table 1:** Cytotoxic effects of heat-killed cells of different LAB strains on cancer cell lines as assessed by the MTT assay.

Cell line	Cytotoxicity (%)							
	LGG		KU22001		KU22002		KU22005	
	8 Log CFU/ml	9 Log CFU/ml	8 Log CFU/ml	9 Log CFU/ml	8 Log CFU/ml	9 Log CFU/ml	8 Log CFU/ml	9 Log CFU/ml
AGS	0.98 ± 1.78^d^	23.75 ± 2.15^b^	2.08 ± 3.73^cd^	23.92 ± 4.95^b^	1.30 ± 1.79^d^	26.09 ± 2.16^b^	7.53 ± 2.34^c^	32.15 ± 4.78^a^
HT-29	3.01 ± 1.28^b^	10.81 ± 2.05^a^	2.04 ± 0.75^b^	9.41 ± 0.96^a^	1.58 ± 0.93^b^	11.08 ± 1.57^a^	1.56 ± 0.73^b^	9.85 ± 1.32^a^
DLD-1	17.97 ± 5.47^c^	27.62 ± 1.67^b^	28.63 ± 3.43^b^	32.67 ± 5.26^b^	34.08 ± 3.58^b^	52.08 ± 3.42^a^	34.90 ± 6.03^b^	46.57 ± 4.82^a^
LoVo	42.43 ± 5.37^b^	46.86 ± 3.66^b^	35.83 ± 2.11^c^	56.18 ± 0.84^a^	25.98 ± 2.45^d^	54.37 ± 1.87^a^	27.14 ± 3.20^d^	59.40 ± 2.86^a^
Caco-2	4.75 ± 0.92^e^	17.43 ± 0.34^cd^	23.07 ± 1.94^b^	41.51 ± 4.89^a^	20.32 ± 4.31^bc^	38.15 ± 2.14^a^	15.08 ± 1.87^d^	39.73 ± 2.85^a^
HeLa	2.68 ± 1.50^f^	10.23 ± 1.49^d^	17.64 ± 2.75^c^	36.22 ± 1.50^a^	12.35 ± 2.99^d^	35.70 ± 1.12^a^	6.41 ± 1.98^e^	27.30 ± 2.73^b^
MCF-7	3.60 ± 1.41^f^	7.54 ± 0.93^e^	24.03 ± 0.49^c^	36.31 ± 1.70^a^	19.05 ± 0.31^d^	29.87 ± 1.62^b^	19.08 ± 0.19^d^	28.89 ± 2.64^b^
A549	7.14 ± 0.78^e^	15.19 ± 2.09^d^	16.59 ± 1.25^d^	26.24 ± 0.31^b^	13.74 ± 2.18^d^	23.61 ± 1.97^bc^	21.13 ± 2.80^c^	36.33 ± 0.90^a^
HepG2	8.21 ± 1.27^e^	28.35 ± 1.44^c^	18.63 ± 1.25^d^	41.92 ± 0.80^b^	17.07 ± 0.86^d^	40.59 ± 0.46^b^	18.57 ± 0.29^d^	44.00 ± 0.44^a^

LGG, *Lacticaseibacillus rhamnosus* GG; KU22001, *Enterococcus faecium* KU22001; KU22002, *Enterococcus faecium* KU22002; KU22005, *Enterococcus faecium* KU22005.

The cell viability data are represented as the mean ± SD derived from three independent experiments.

Different letters above the value indicate significant differences for each characteristic (*p* < 0.05).

**Table 2:** Cytotoxic effects of 9 log CFU/ml of heat-killed LAB strains on HeLa cells assessed using the MTT assay.

LAB	Cytotoxicity (%)			
	37°C, 20 h	37°C, 40 h	37°C, 60 h	25°C, 20 h
LGG	10.23 ± 1.49^c^	4.65 ± 1.84^c^	4.16 ± 1.48^c^	31.35 ± 1.01^b^
KU22001	36.22 ± 1.50^a^	29.69 ± 1.11^a^	27.85 ± 1.80^a^	39.93 ± 1.96^a^
KU22002	35.70 ± 1.12^a^	27.85 ± 0.43^a^	28.92 ± 2.53^a^	37.97 ± 1.71^a^
KU22005	27.30 ± 2.73^b^	25.06 ± 0.45^b^	20.40 ± 1.43^b^	33.72 ± 0.79^b^

LGG, *Lacticaseibacillus rhamnosus* GG; KU22001, *Enterococcus faecium* KU22001; KU22002, *Enterococcus faecium* KU22002; KU22005, *Enterococcus faecium* KU22005.

The cell viability are represented as the mean ± SD derived from three independent experiments.

Different letters of superscripts indicate significant differences (*p* < 0.05).

**Table 3:** Effect of temperature on EPS production by LAB strains grown at 37°C and 25°C.

LAB	37℃ (mg EPS/ml)	37℃ (mg EPS/9 log CFU)	25℃ (mg EPS/ml)	25℃ (mg EPS/9 log CFU)
LGG	0.617 ± 0.051^c^	0.123 ± 0.010^c^	1.421 ± 0.122^c^	0.497 ± 0.043^c^
KU22001	1.189 ± 0.104^a^	1.133 ± 0.099^a^	1.974 ± 0.047^a^	1.880 ± 0.044^a^
KU22002	1.200 ± 0.048^a^	1.143 ± 0.046^a^	1.899 ± 0.087^a^	1.808 ± 0.082^a^
KU22005	1.039 ± 0.096^b^	0.990 ± 0.092^b^	1.703 ± 0.039^b^	1.622 ± 0.037^b^

LGG, *Lacticaseibacillus rhamnosus* GG; KU22001, *Enterococcus faecium* KU22001; KU22002, *Enterococcus faecium* KU22002; KU22005, *Enterococcus faecium* KU22005.

The EPS production are expressed as the mean ± SD derived from three independent experiments.

Different letters of superscripts indicate significant differences (*p* < 0.05).

EPS concentratio*n* = (OD at 490 nm – 0.4969)/0.0021 (R^2^ = 0.995)
