# Supplementary material for: Heat-Killed Enterococcus faecium KU22001 Having Effective Anti-Cancer Effects on HeLa Cell Lines at a Lower Temperature
Source: J Microbiol Biotechnol. 2024 Jan 8;34(4):902–10. doi: 10.4014/jmb.2310.10050 (PMC11091667; doi:10.4014/jmb.2310.10050)
Supplement: Supplementary file 1 [file jmb-34-4-902-supple.pdf]

J. Microbiol. Biotechnol. 2024. 34(4): 902–910
https://doi.org/10.4014/jmb.2310.10050

Supplementary Tables and Figure

Table 1S. List of primer sequences used for semi-quantitative RT-PCR.

Primer		Sequence (5′ to 3′)
β-Actin	(Forward)	TTCTGACGGCAACTTCAACT
	(Reverse)	GTCCAGCCCATGATGGTTCT
Bax	(Forward)	TCACCCTGAAGTACCCCATC
	(Reverse)	GTCCAGCCCATGATGGTTCT
Bcl-2	(Forward)	CAGCTGCACCTGACGCCCTT
	(Reverse)	GCCTCCGTTATCCTGGATCC
Caspase-3	(Forward)	TTTGTTTGTGTGCTTCTGAGCC
	(Reverse)	ATTCTGTTGCCACCTTTCGG
Caspase-9	(Forward)	TGCTGCGTGGTGGTCATTCTC
	(Reverse)	CCGACACAGGGCATCCATCTG

β-Actin, housekeeping gene; *Bax*, *Bcl-2* associated X protein, apoptosis regulator; *Bcl-2*, B cell lymphoma, apoptosis regulator; *caspase-3*, effector caspase, interactor with caspase-8 and caspase-9; *caspase-9*, initiator caspase, critical for the apoptotic pathway.

Table 2S. Viability of MRC-5 cells treated with heat-killed cells of LAB strains detected using the MTT assay.

Cell line	Cell viability (%)							
	LGG		KU22001		KU22002		KU22005	
	8 Log CFU/ml	9 Log CFU/ml	8 Log CFU/ml	9 Log CFU/ml	8 Log CFU/ml	9 Log CFU/ml	8 Log CFU/ml	9 Log CFU/ml
MRC-5	100.69 ± 3.89^{d}	107.43 ± 6.33^{bc}	103.31 ± 3.69^{cd}	114.23 ± 1.83^{a}	114.41 ± 2.50^{a}	116.02 ± 1.63^{a}	112.74 ± 2.90^{ab}	102.89 ± 2.05^{cd}

LGG, *Lacticaseibacillus rhamnosus* GG; KU22001, *Enterococcus faecium* KU22001; KU22002, *Enterococcus faecium* KU22002; KU22005, *Enterococcus faecium* KU22005.

The data of cell viability are expressed as the mean ± SD derived from three independent experiments.

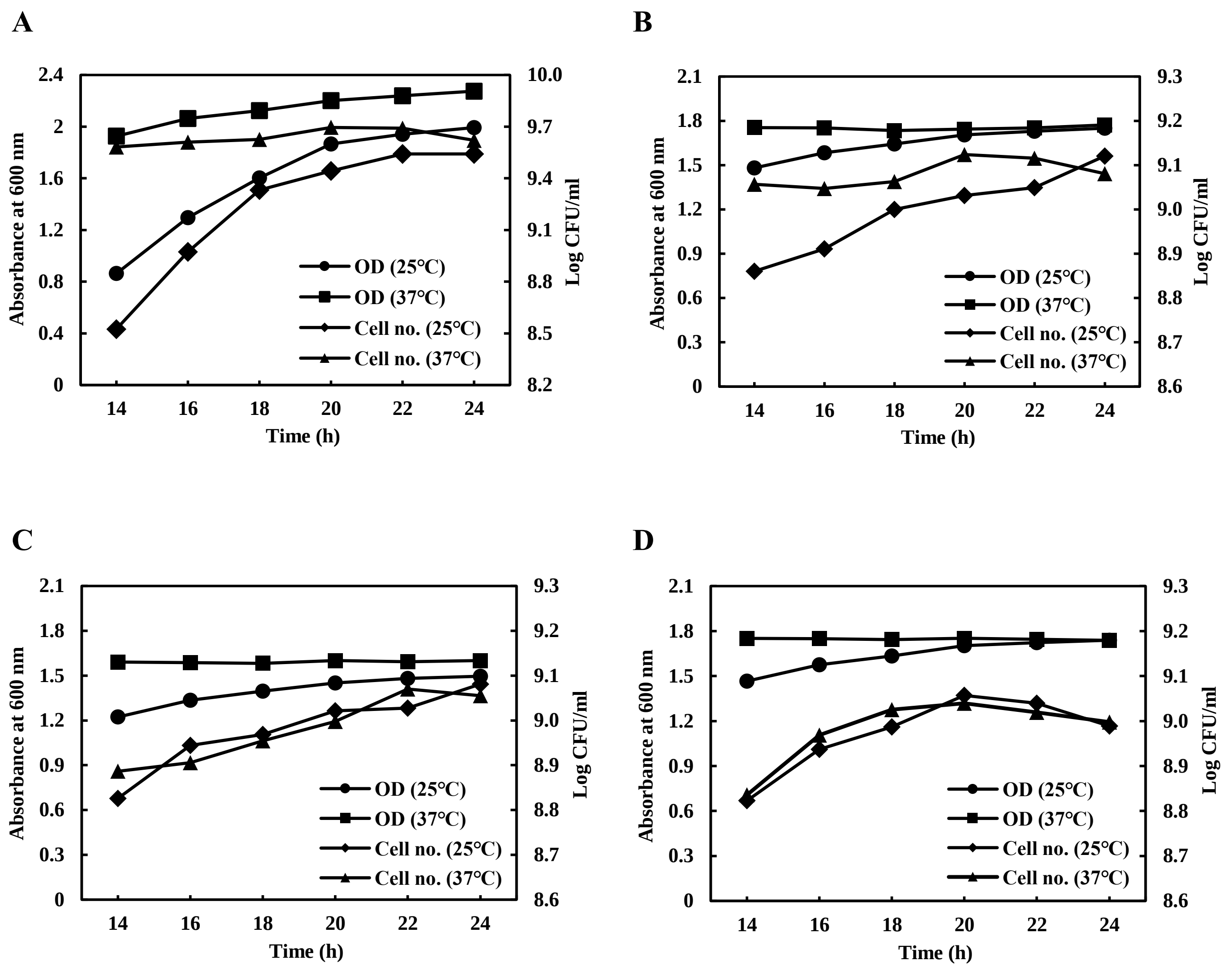

Fig. 1S. Growth of LGG and *E. faecium* in MRS at 25°C and 37°C. (**A**) LGG, (**B**) KU22001, (**C**) KU22002, and (**D**) KU22005.

LGG, *Lacticaseibacillus rhamnosus* GG; KU22001, *Enterococcus faecium* KU22001; KU22002, *Enterococcus faecium* KU22002; KU22005, *Enterococcus faecium* KU22005.
